# Editorial: Bioleaching and Biocorrosion: Advances in Interfacial Processes

**DOI:** 10.3389/fmicb.2021.653029

**Published:** 2021-03-17

**Authors:** Ruiyong Zhang, Jizhou Duan, Dake Xu, Jinlan Xia, Jesús A. Muñoz, Wolfgang Sand

**Affiliations:** ^1^Key Laboratory of Marine Environmental Corrosion and Bio-Fouling, Institute of Oceanology, Chinese Academy of Sciences, Qingdao, China; ^2^Open Studio for Marine Corrosion and Protection, Pilot National Laboratory for Marine Science and Technology (Qingdao), Qingdao, China; ^3^Center for Ocean Mega-Science, Chinese Academy of Sciences, Qingdao, China; ^4^Shenyang National Laboratory for Materials Science, Northeastern University, Shenyang, China; ^5^School of Minerals Processing and Bioengineering, Central South University, Changsha, China; ^6^Department of Chemical and Materials Engineering, Complutense University of Madrid, Madrid, Spain; ^7^Textile Pollution Controlling Engineering Center of Ministry of Environmental Protection, College of Environmental Science and Engineering, Donghua University, Shanghai, China; ^8^Aquatic Biotechnology, University of Duisburg-Essen, Essen, Germany; ^9^Institute of Biosciences, Freiberg University of Mining and Technology, Freiberg, Germany

**Keywords:** bioleaching, microbially influenced corrosion, attachment, biofilms, extracellular polymeric substances, fluorescence microscopy, bioinformatics

The adherence of microbes to surfaces is essential for optimal living conditions including protection against external influences such as temperature, pH, or even biocidal agents. The resultant surface growth of microorganisms and their metabolic products, extracellular polymeric substances (EPS) – “the dark matter” termed by Thomas Neu – form biofilms, which tend to coat virtually every material surface in contact with water (Flemming et al., 2016). The interaction of microorganisms in the form of biofilms with mineral/metallic surfaces emerges two dissolving processes of materials: microbiologically influenced corrosion (MIC) and bioleaching. The attachment of microorganisms to surfaces and the involved EPS play a pivotal role in the biocorrosion/biodeterioration of materials (Ma et al., 2020) and bioleaching of various minerals (chiefly metal sulfide ores) (Sand and Gehrke, 2006; Zhang et al., 2016). Both MIC and bioleaching, are processes characterized by the dissolution of substrata (controlled and uncontrolled).

The two well-established bioleaching modes are “non-contact” and “contact” leaching (Vera et al., 2013). The latter takes into account that cells attach and form biofilms on the surface of minerals. During bioleaching of ores, the formation of various redox reaction products and mineral phase transformations and changes in the microbial community structure takes place. These changes modify the interfacial interactions defining the bioleaching behavior and metal extraction efficiency.

MIC, the deterioration of materials such as steel and concrete, is the result of electro/chemical reactions influenced or driven by microorganisms, which are often present as biofilms. In recent years it has become clear that microbes/biofilms not only cause material corrosion, they can also inhibit or protect it against corrosion through microbiologically influenced corrosion inhibition (MICI) (Nagiub and Mansfeld, 2001). The MIC efficiency is the result of the activity of diverse microbial species in biofilms and by different environmental conditions, along with multiple types of interfacial media, and by the materials composition and surface characteristics. Furthermore, MIC by electro-active microbes occurs as part of an extracellular electron transfer (EET) – MIC (Dinh et al., 2004; Kato, 2016; Li et al., 2018). To date, there are still research gaps in the field of MIC, as pointed out in the recent review by Little et al. (2020).

This Research Topic comprises 17 original research articles and presents recent progress in bioleaching and MIC, especially the interactions between minerals/materials with dissolving/corroding-microorganisms. Included are nine papers on MIC and eight papers on bioleaching. The focus on the corrosive behavior of the halophilic archaeon *Natronorubrum (N.) tibetense* was investigated by Qian et al. The results indicate that increased dissolved oxygen concentration (DOC) in the range of <3 ppm promoted cell growth and cathodic corrosion, causing increased MIC. While after reaching 5 ppm, the increased proportion of biofilm on the surface of carbon steel enhanced the inhibition effect of the oxygen diffusion. Consequently, the MIC of carbon steel is weaker than that in 3 ppm DOC inoculated medium. The biofilms of *N. tibetense* showed a significant influence on MIC behavior via influencing the oxygen concentration.

A corrosion model of Q235 carbon steel in mixed cultures of *Mariprofundus ferrooxydans* and *Thalassospira* sp. is proposed by Chen et al. This study reveals that biofilms cause varied surface characteristics such as electrochemical heterogeneity and induce or accelerate localized corrosion. Liu et al. show that *Desulfotomaculum nigrificans* increase under deposit corrosion (UDC) by increasing anodic and cathodic reactions, heterogenous biofilms were shown to promote localized corrosion as reported for other sulfate-reducing microbial strains. The studies by Hu et al. on corrosion of printed circuit boards with an immersion silver layer (PCB-ImAg) observed the acceleration of microporous corrosion caused by *Bacillus cereus* metabolites. Electrochemical impedance spectroscopy (EIS) tests confirmed that the MIC of PCB-ImAg is related to biofilm formation and microbial metabolism. The work by Wu et al. also indicates that biofilm development and acid production can spontaneously influence steel corrosion, highlighting the concept of multi MIC mechanisms.

The strategy of using biofilm-induced biomineralization to protect corrosion seems to be effective and shields light on green technology for corrosion protection (Liu et al., 2018). Studies by Guo et al. demonstrate that the biomineralized films induced by biofilms of certain microorganisms are a promising approach for mitigation of the corrosion of steel in a complex microbial environment. Liu et al. propose a strategy by using mixtures of amino acids to alleviate microbial corrosion by *Vibrio harveyi* biofilm in aircraft fuel tanks. An et al. developed a versatile multiport flow test column for accurate MIC monitoring which contributes to the standardization of MIC tests in the laboratory. This column system demonstrates that higher corrosion is caused by methanogenic archaea than sulfate-reducing bacteria under certain conditions. Zhang et al. used metagenomic sequencing and analyzed microbial communities in copper-associated biofilms in coastal seawater. The authors observed abundant copper-resistance genes plus genes related to EPS production, indicating the presence of diverse copper-resistance patterns.

In another contribution to this Research Topic, Blazevic et al. studied the interactions of the thermoacidophilic archaeon *Metallosphaera sedula* with the tungsten mineral scheelite. They suggest a biooxidation pretreatment for processing tungsten ores, which contributes to the understanding of the biogeochemistry of tungsten in natural environments. By using X-ray photoelectron spectroscopy (XPS) and microscopic techniques Yin et al. investigated the microbial-arsenopyrite interactions and propose dissolution pathways for a mixed culture of acidophiles. Huynh et al. studied the attachment, biofilm formation of *Sulfobacillus (Sb.) thermosulfidooxidans* on pyrite in the presence of chloride, which has implications for bioleaching using seawater. Chaerun et al. describe the extraction of Pb from Indonesian galena concentrate by using an iron- and sulfur-oxidizing mixotrophic bacterium *Citrobacter* sp.

The study by Li et al. provides insight into the interaction patterns of *Leptospirillum (L.) ferriphilum* with *Sb. thermosulfidooxidans*. The authors point out the importance of the introduction time of *Sb. thermosulfidooxidans* in a short-term leaching system. Liu et al. investigated the introduction of *Acidithiobacillus (At.) ferrooxidans* on the bioleaching efficiency of low-grade copper sulfide by the acidophilic consortia. The data indicate sophisticated microbial interactions during the bioleaching process and provide a basis for a strategy to improve bioleaching in practice. Saavedra et al. show that a suitable amount of galactose can promote EPS production by *At. ferrooxidans* and, thus, increase the microbial tolerance to ferric ions. This finding offers a base for strategic design, such as the pretreatment of cells in biohydrometallurgical processes.

The article by Vardanyan et al. describes the molecular details of extracellular carbohydrates of a newly isolated strain of *At. ferrooxidans* Ksh. Exosaccharide produced by isolate Ksh is present mainly as polysaccharide in contrast to *L. ferriphilum* CC, which is oligosaccharide. The degree of hydration of the saccharide molecules causes the structural difference of colloidal particles of these polysaccharides.

For improved control of both, bioleaching and MIC processes, an understanding of the interactions of biofilms with minerals/materials is of crucial importance. The articles in this Research Topic share important information on how material-dissolving microorganisms interact with minerals/metals, providing insights into the underlying mechanisms of microbial interactions with substratum/substrate surfaces. These studies indicate strategies to control bioleaching (promote) and MIC (mitigate) processes by interfering or controlling biofilm microenvironments between these specific groups of microorganisms and material surfaces. The subject of interfacial sciences in the field of bioleaching and biocorrosion, examining areas such as the mechanisms of interfacial interactions between leaching microorganisms and minerals or biocorrosion microorganisms and metals/construction materials, microbial interactions (quorum-sensing mechanisms), and electron transfer pathways within the biofilm microenvironments, will doubtless continue to draw attention and fascinating outcomes are expected once advanced microscopy techniques such as cryogenic electron microscopy (Cryo-EM), omics techniques like metagenomic, proteomics, and gene manipulation are combined, advancing this multi-interdisciplinary topic.

## Author Contributions

All authors listed have made a substantial, direct and intellectual contribution to the work, and approved it for publication.

## Conflict of Interest

The authors declare that the research was conducted in the absence of any commercial or financial relationships that could be construed as a potential conflict of interest.
